# Heavy Metal Contamination of Soil in Preschool Facilities around Industrial Operations, Kuils River, Cape Town (South Africa)

**DOI:** 10.3390/ijerph19074380

**Published:** 2022-04-06

**Authors:** Busisiwe Shezi, Renée Anne Street, Candice Webster, Zamantimande Kunene, Angela Mathee

**Affiliations:** 1 Environment and Health Research Unit, South African Medical Research Council, Johannesburg 2094, South Africa; candice.webster@mrc.ac.za (C.W.); zama.kunene@mrc.ac.za (Z.K.); angie.mathee@mrc.ac.za (A.M.); 2 Department of Environmental Health, Faculty of Health Sciences, University of Johannesburg, Johannesburg 2094, South Africa; renee.street@mrc.ac.za; 3 Environment and Health Research Unit, South African Medical Research Council, Durban 4001, South Africa; 4 Department of Environmental Health, School of Behavioural and Lifestyle Sciences, Faculty of Health Sciences, Nelson Mandela University, Port Elizabeth 6019, South Africa

**Keywords:** health risk assessment, technogenic soils, SUITMAs, environmental health, children, buffer zones, factor analysis

## Abstract

The contamination of soil by heavy metals is a potential health risk, especially among susceptible populations. The aim of this study was to measure the levels of heavy metals, identify the contamination levels and possible sources of heavy metals, and evaluate the health risk caused by heavy metals to the children living in Kuils River. Composite samples of soil were collected at 34 preschools. A portable X-ray fluorescence spectrometer was used to measure the levels of metals. Contamination levels were evaluated using a geoaccumulation index (Igeo), enrichment factor (EF), contamination factor (CF) and pollution load index (PLI). The spatial distribution of the Igeo contamination levels was assessed using ArcGIS. Sources of heavy metals and the correlation among metals were assessed using factor analysis and Pearson correlation, respectively. The measured concentrations of metals were used to estimate the health risk for children. The average levels of the metals were 16, 4469, 137, 30, 176, 1547 and 232 mg/kg for arsenic (As), iron (Fe), manganese (Mn), lead (Pb), strontium (Sr), titanium (Ti) and zinc (Zn), respectively. According to Igeo, EF, CF and PLI contamination exist in the study area. The health index (HI) for non-carcinogenic effects showed the ingestion route as the main contributor to the total risk, with the accumulative carcinogenic risk exceeding the maximum acceptable level. To protect the affected communities, and children in particular, this study provides evidence of the need for action, including the institution of mandatory buffer zones between pollutant-generating activities and human settlements.

## 1. Introduction

Poor town planning practices, industrial growth and urbanization have led to the location of various industrial processes such as mining operations, battery manufacturing and recycling and smelting operations within or in close proximity to residential areas [1,2,3,4]. The emission of pollutants such as heavy metals into air, water and soil may cause significant local and downstream contamination, as well as harmful exposures in affected communities [5,6,7,8].

Heavy metal exposure may result in a range of adverse health impacts, including neurocognitive effects, behavioral disorders, cardiovascular diseases and respiratory problems [9,10,11], even at very low levels. Such health issues are exacerbated in vulnerable groups. For example, prenatal exposure to metals has been reported to result in significant negative effects on mental (−2.51, 95% CI: −4.92, −0.10) and psychomotor (−2.93, 95% CI: −6.00, 0.13) development indices of children at six months [10]. Similarly, significant positive associations between blood chromium (Cr) with cough (OR: 1.91; 95% CI: 1.17, −3.13) and blood lead > 5 μg/dL with asthma (OR: 9.50; 95%CI: 1.16–77.49) have also been reported in children [11].

Human exposure to heavy metals occurs through, for example, inhalation of dust particles, consumption of contaminated crops or produce from residential food gardens and drinking contaminated water [12,13]. Children are particularly vulnerable to ingestion of heavy metals (hand-to-mouth and object-to-mouth exposure pathways) deposited in soil [14]. Ingestion of soil or dust as a result of hand-to-mouth activity has been shown to account for approximately 50% of the lead (Pb) intake in children, [15] and thus a significant association between soil Pb levels and blood Pb levels of children is reported in the literature [14].

Soils are known to influence human health as well as perform a variety of ecosystem services such as acting as a basis for plants, food production and raw materials [16,17,18]. When changed from their natural state or impacted on by pollution, soils are unable to perform these services or maintain functional ecosystems [19]. Studies in China [20], Mexico [21], Uganda [1] and Bangladesh [22] have assessed soil contamination in communities situated in close proximity to industrial operations and reported levels exceeding guidelines for environmental soil contamination. In South Africa, elevated levels of heavy metals exceeding the South African and Canadian reference levels have also been shown. For example, Kapwata et al. [6] reported elevated levels of arsenic (As) with 54% of household garden soil samples exceeding the Canadian reference level of 20 mg/kg in a rural setting. A further study by Mathee et al. [7] reported elevated levels of Pb in soil of the homes situated near mine dumps, with 6.5% of samples exceeding the South African reference level of 230 mg/kg and 13% exceeding the Canadian reference level of 120 mg/kg [7]. Young children spend a considerable portion of their time in preschool facilities, often involved in play and developmental activities in their indoor and outdoor environments. Therefore, children who live or attend school in close proximity to industrial developments, especially where buffer zones are inadequate or absent, or where implementation of regulations and standards is lax, may become exposed to highly elevated concentrations of heavy metals in soil. Human health risk assessment studies conducted to estimate the health effects that may result from exposure to carcinogenic and non-carcinogenic heavy metals reveal a cause for concern [23]. For example, Kamunda et al. [24] estimated a hazard index (HI) value > 1 (which poses serious non-carcinogenic effects) among children living in the gold mining area, Johannesburg, South Africa. The carcinogenic risk was found to be 3.67 × 10^−4^, implying that of 2725 children, one child may be affected [24].

Kuils River is a residential suburb located around 25 km east of central Cape Town, South Africa. Alongside residential homes, Kuils River also has commercial, industrial and agricultural zones. For example, there is a steel mill in Kuils River that has caused a public outcry over emissions and concerns over health effects [25].

The main objectives of this study were to (i) measure the levels of heavy metals in preschool facilities situated around an industrial zone in Kuils River, Cape Town, (ii) evaluate the contamination levels of heavy metals relative to geological background levels using a geo-accumulation index, enrichment factor, contamination factor and pollution load index, (iii) assess the spatial distribution of the contamination levels in relation to the industrial zone using ArcGIS, (iv) identify the possible sources of heavy metals using multivariate statistical techniques and (v) evaluate the health risk caused by heavy metals to the children living in Kuils River.

## 2. Materials and Methods

### 2.1. Study Area and Sample Collection

This was a descriptive, cross-sectional study undertaken in November 2020, in the city of Cape Town, Western Cape province, South Africa. The Western Cape has a Mediterranean-type climate, and the geology is dominated by pre-Cambrian metamorphosed shales, sedimentary and igneous rocks [26]. The study was conducted in the suburb of Kuils River, which has a population of ±47,000 people [27]. The climate of the Kuils River is generally influenced by the south Atlantic anti-cyclone and therefore in a southeasterly wind regimen [28]. The topography varies from steep mountain ridges to level ground. Soils are sandy, acidic and deficient in nutrients, and the vegetation is naturally fynbos [29]. The study area is highly urbanized with extensive commercial agriculture and industrial operations, including iron and steel manufacturers, oil refineries and paint manufacturers.

**Figure 1:**
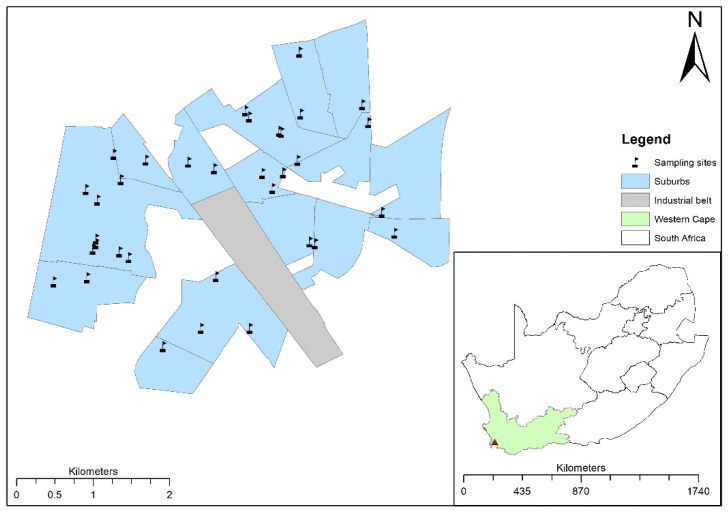
Location of sampled preschools in relation to the industrial zone.

For the current study, all preschool facilities (*n* = 50) situated within 5 km from the industrial zone were targeted. There was a 68% response rate in this study; the reasons for non-inclusion included refusal to participate due to fear of being infected with COVID-19, the preschool owner not being available and construction being underway at the time of the visit. Some of the preschools were closed due to COVID-19 restrictions. Therefore, 34 preschools within a radius of 2.5 km from the industrial belt were assessed. The sampling grid for the assessed preschools (*n* = 34) is shown in Figure 1. For anonymity, each preschool was assigned an ID code.

Soil samples from preschool institutions, mainly from the garden area, children’s play area or from the roadside (adjacent to or opposite the preschool) were collected into sample jars using a stainless-steel spatula. Five samples of topsoil (10 cm) were taken per site, and then mixed thoroughly to create a composite sample using the guidelines set by the US EPA [30]. Sampling points were recorded using a portable global positioning system (GPS Garmin eTrex 20) (Garmin, Olathe, KS, USA) which was verified using Google Maps.

### 2.2. Sample Preparation and Analysis

The samples were oven-dried at 40 degrees Celsius, thoroughly mixed and sieved to particles less than 2 mm. A portable X-ray fluorescence (XRF) spectrometer was used to measure the levels of arsenic (As), iron (Fe), manganese (Mn), lead (Pb), strontium (Sr), titanium (Ti) and zinc (Zn). Prior to and during soil analysis, the XRF machine was calibrated following the guidelines set by the manufacturer (Thermo Fisher Scientific, Waltham, MA, USA). The accuracy was checked on duplicate samples, and each sample was analyzed for 60 s. Results of soil analyses were checked against South African and Canadian guidelines [31,32].

### 2.3. Statistical Analysis

All the data were cleaned and checked for errors in Microsoft Excel and then exported to Stata IC version 14 (StataCorp, College Station, TX, USA) for further analysis. Quality control measures to maintain data integrity included removing duplicates and correcting data capture errors before coding variables. Descriptive statistics such as means (SD), medians and ranges were calculated. To evaluate the contamination levels of heavy metals in soil, the geo-accumulation index (Igeo), enrichment factor (EF), contamination factor (CF) and pollution load index (PLI) were used [22,33,34]. Igeo was computed using the following equation:

(1) $$\mathrm{Igeo}\;={\mathrm{Log}}_{2}\left(\frac{\mathrm{Cn}}{1.5\;\times \;\mathrm{Bn}}\right)$$

where Cn is the measured concentration of the element ‘n’ and Bn is the geochemical background value of the same element. The background values for the study area were based on Turekian and Wedepohl [35]. The constant 1.5 was introduced to address the possible deviations in background concentration of heavy metals, which may be associated with lithogenic and anthropogenic effects. The Igeo scores range from 0 (uncontaminated) to 6 (extremely contaminated) relative to the background concentration [34]. Enrichment factor was calculated using the following equation:

(2) $$\mathrm{EF}=\frac{\left(\mathrm{Metal}/\mathrm{RE}\right)\mathrm{sample}}{\left(\mathrm{Metal}/\mathrm{RE}\right)\mathrm{background}}$$

where ((Metal/RE) sample) is the value of the metal levels assessed, and ((Metal/RE) background) is the geochemical background value of the same element. Values of 0.5 ≤ EF ≤ 1.5 suggest that the metal concentration may come entirely from natural weathering processes [25], and an EF > 1.5 indicates that a significant portion of the metals was delivered from human activities [36]. Contamination factor was calculated by using the metal levels divided by the geochemical background value, and PLI [37] is expressed as follows:

(3) $$\mathrm{PLI}=\left(\mathrm{CFAs}\;\times \mathrm{CFFe}\;\times \mathrm{CFMn}\;\times \mathrm{CFPb}\;\times \mathrm{CFSr}\;\times \mathrm{CFTi}\;\times \mathrm{CFZn}\right)\;1/7$$

where CF is the contamination factor obtained by calculating between each metal’s concentration and its background value.

GIS mapping techniques were undertaken to produce spatial distribution maps of contamination index levels in the study area. Geographic base maps of suburbs within the City of Cape Town in shapefile format were acquired from the City of Cape Town’s Open Data Portal. The sampling points were imported into ArcMap and symbolized according to the contamination index level. Local spatial correlation (characterized by Getis-Ord Gi* hot spot analysis) was performed to identify whether features with high values (hot spots) or features with low values (cold spots) tend to cluster in the study area. The output of Gi* statistics is a Z-score. The larger the Z score (for statistically significant positive Z scores), the more intense the clustering of high metal concentrations, and the smaller the Z score (for statistically significant negative Z scores), the more intense the clustering of low metal concentrations. All maps were produced using ArcGIS 10.6.1.V(ESRI, Durban, South Africa) [38].

Factor analysis and Pearson correlation analysis were used to identify the common relationship of heavy metals that may indicate a similarity in source. In this study, the heavy metal levels were log-transformed prior to multivariate analysis. Bartlett’s test was used to determine the appropriateness of factor analysis [39]. Principal factor and varimax rotation techniques were used to determine the relationship between factors. The factors with an eigenvalue ≥ 1 (total variance accounted for by each factor) were retained.

Health risks for children were identified by measuring average daily intake (ADI) of heavy metals through ingestion, inhalation and dermal contact. ADI (mg/kg per day) was calculated using the following equations [40]:

(4) $${\mathrm{ADI}}_{\mathrm{ingest}}=\frac{C\;\times \;\mathrm{IngR}\;\times \;\mathrm{EF}\;\times \;\mathrm{ED}}{\mathrm{BW}\;\times \;\mathrm{AT}}$$

(5) $${\mathrm{ADI}}_{\mathrm{inh}}=\frac{\mathrm{Cs}\;\times \;\mathrm{InR}\;\times \;\mathrm{EF}\;\times \;\mathrm{ED}}{\mathrm{BW}\times \;\mathrm{AT}\;\times \;\mathrm{PEF}}$$

(6) $${\mathrm{ADI}}_{\mathrm{dems}}=\frac{\mathrm{Cs}\;\times \;\mathrm{SA}\;\times \;\mathrm{FE}\;\times \;\mathrm{AF}\;\times \;\mathrm{ABS}\;\times \;\mathrm{EF}\;\times \;\mathrm{ED}\;\times \;\mathrm{CF}}{\mathrm{BW}\;\times \;\mathrm{AT}}$$

where C is the metal concentration in soil (mg/kg), IngR is the ingestion rate in mg/day (IngR = 200 mg/day), EF is exposure frequency in days/year (350 days/year), ED is exposure duration (6 years), BW is the average birth weight as 15 kg, InhR is inhalation rate in m^3^/day, average time for carcinogens (365 × 70 days), average time for non-carcinogens (365 × ED days), PEF is the particle emission factor in m^3^/kg (1.3 × 109), SA is the surface area of the exposed skin in cm^2^ (2100 cm^2^), FE is the dermal exposure ratio (0.61), AF is the skin adherence factor for the soil in mg/cm^2^ (0.2 mg/cm^2^), ABS is the dermal absorption factor (0.1) and CF is the conversion factor in kg/mg (10^−6^ kg/mg).

The hazard quotient was calculated to estimate the potential non-carcinogenic risk of heavy metals to children using the following equation [41]:

(7) $$\mathrm{HQ}=\frac{\mathrm{ingestion},\;\mathrm{inhalation}\;\mathrm{or}\;\mathrm{dermal}}{\mathrm{RfD}}$$

where HQ is the ratio of ADI (from three exposure pathways) and RfD is the chronic reference dose for each heavy metal in mg/kg/day. RfDs for metals are shown in Table 1:

The incremental probability of an individual developing cancer over a lifetime as a result of exposure to potential carcinogens was estimated using the following equation:

(8) HI=∑HQ=HQing + HQinh + HQderm

where HI is the hazard index defined as the sum of HQ (ingestion + inhalation + dermal). HI ≤ 1 indicates no significant risk of non-carcinogenic effect and HI > 1 indicates significant risk of non-carcinogenic effect.

For each exposure pathway, cancer risk for lifetime exposure (LCR) was estimated to determine the risk by cumulative life cancer risk rating (CSF is the cancer slope factor which is shown in Table 1) using the following equation:

(9) $$\mathrm{LCR}=\mathrm{ADI}\left(\mathrm{ingestion},\;\mathrm{inhlation}\;\mathrm{or}\;\mathrm{dermal}\right)\times \;\mathrm{CSF}$$

## 3. Results

A total of 143 samples were collected from 34 preschools. Table 2 shows descriptive statistical data for the concentration of heavy metals in the soil samples. The mean (SD) levels for As, Fe, Mn, Pb, Sr, Ti and Zn were 16 mg/kg (±17), 4469 mg/kg (±3093), 137 mg/kg (±40), 30 mg/kg (±18), 176 mg/kg (±16), 1547 mg/kg (±765) and 232 mg/kg (±123), respectively.

The concentrations of As, Mn, Pb and Zn in all samples were below the South African reference levels [24], but 31% (As) and 9% (Zn) of samples had heavy metal levels exceeding the more protective Canadian reference levels (reference levels for Fe, Sr and Ti are not available) [23]. Levels of As (36%), Fe (50%), Mn (31%), Pb (22%), Sr (33%), Ti (38%) and Zn (21%) were above the total sample mean heavy metal level (Table 1).

According to Igeo results, all the preschools were relatively contaminated by As and Zn, 21 preschools were relatively contaminated by Pb and 30 preschools were relatively contaminated by Sr. None of the assessed preschools were contaminated by Mn, Fe or Ti. EFs > 1.5 were obtained for the elements As (6.2 < EF <18.3) and Pb (<1.6 EF < 9.2) in all the preschools. EFs < 1.5 were obtained for Mn and Fe in all the preschools indicating that they mostly originate from crustal contribution. As for the other elements, a 1.6 < EF < 5.9 was obtained in five preschools, 1.6 < EF < 1.9 was obtained in four preschools and 1.6 < EF < 27.8 was obtained in 30 preschools for Zn, Ti and Sr, respectively. The use of the PLI index to evaluate mutual contamination effects indicated that 21 preschools were contaminated.

**Figure 2:**
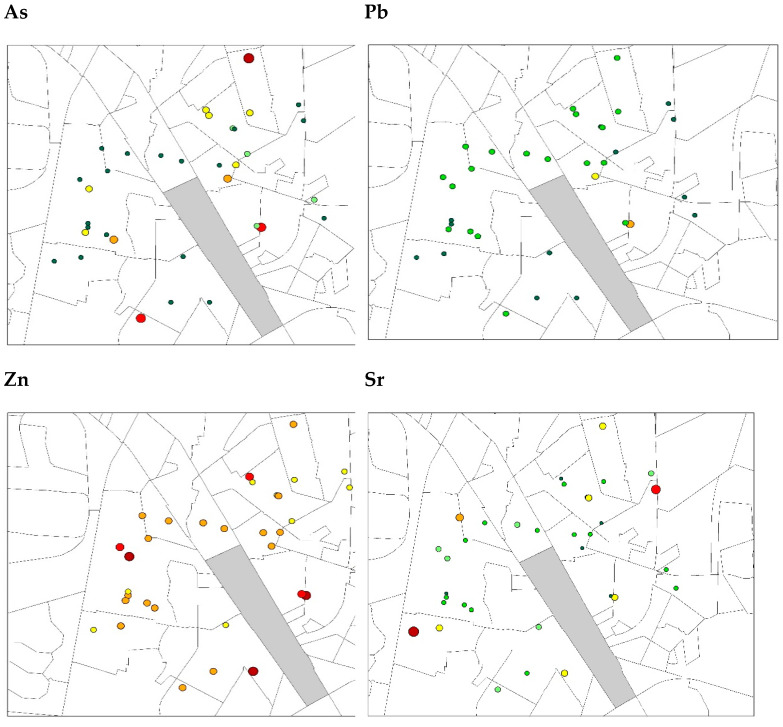

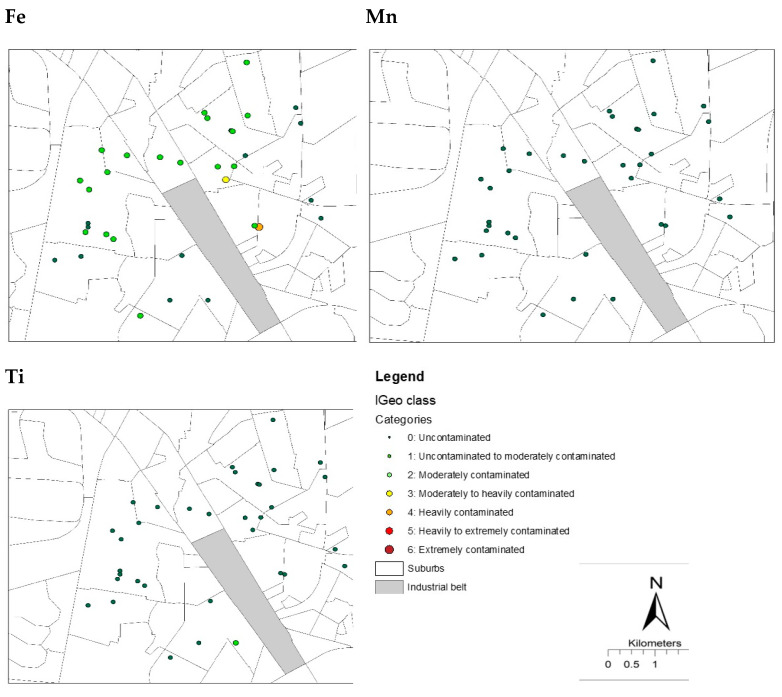
Geo-accumulation Index using mean levels of heavy metals in relation to the industrial zone.

Hot spots for all heavy metals were predominantly situated in the eastern and western parts of the industrial zone (Table 3, Figure 2).

### Source Identification Based on Factor Analysis

Factor analysis retained five factors, and the heavy metals were well represented by the first two factors, having eigenvalues of ≥1. The first two factors accounted for over 80% of the total variance. The values of the loadings as well as cumulative percentage of variance are displayed in Table 4. As, Mn and Sr were highly loaded on Factor 1 and explained 61% of the total variance, and Pb, Fe and Zn were highly loaded on Factor 2 and explained 22% of the total variance.

Significant positive correlations were found at the 0.001 level between As–Fe (0.75), Fe–Ti (0.83), Pb–Fe (0.68), Pb–Zn (0.68), Ti–Zn (0.64) and Fe–Zn (0.53); at the 0.01 level for As–Mn (0.75) and As–Ti (0.62); and at the 0.05 level for As–Sr (0.51), Mn–Sr (0.53) and Pb–Ti (0.45) (Table 5).

HQ for non-carcinogenic effects showed the ingestion route as the main contributor to the total risk, with the accumulative carcinogenic risk exceeding the maximum acceptable level (Table 6).

## 4. Discussion

The findings of this study provide evidence of soil contamination in or around some of the preschool institutions included in the project. Although metal concentrations in soil samples were below the South African reference levels [32], at certain preschools As and Zn concentrations were found to exceed Canadian soil reference levels [45]. The Igeo analyses indicate that contamination has occurred in the study area, while the factor analysis and Pearson correlation coefficient point to the potential roles of industrial operations and natural sources for Factor 1 (As–Mn–Sr), and industrial operations, non-exhaust vehicle emissions (i.e., tire wear, braking system) and natural sources for Factor 2 (Pb–Fe–Zn) [1,46,47,48]. The health index (HI) for non-carcinogenic effects showed the ingestion route as the main contributor to the total risk, with the accumulative carcinogenic risk exceeding the maximum acceptable level. Similar results were found in previous studies for soil conducted in urban communities [24,44,47].

Soils found in anthropized settings such as urban, mining and industrial areas support a diverse array of activities, from supporting buildings and infrastructure to being used for waste management and even urban agriculture [18]. These activities may change the composition and function of soils from their natural state as they are impacted by inorganic contaminants such as heavy metals. The soil samples at Kuils River can be generally classified as loamy sand, consisting of 70% to 85% sand and 10% to 15% clay, and are characterized as loose and gritty, with limited water and nutrient holding capacity. Sandy soils are often acidic. Heavy metals are more soluble in acids, and therefore acidification may increase bioaccumulation of heavy metals and result in increased exposure.

The levels of heavy metals from preschool institutions in the current study were elevated relative to findings from certain studies conducted in urban settings situated in low to middle socio-economic status communities, with a consequent associated risk for children via the soil–hand–mouth pathway [49]. The average As levels (16 mg/kg) reported in this study are in the range reported in residential soil studies conducted in other parts of South Africa, such as Limpopo Province (0.2–25 mg/kg) [6] and the city of Johannesburg (0.1–65) [7]. On the other hand, the average Mn (137 mg/kg), Pb (30 mg/kg), Sr (176 mg/kg) and Zn (232 mg/kg) levels were much higher than the levels reported in mine tailings of Johannesburg; Mn (43 mg/kg), Pb (17 mg/kg), Sr (2 mg/kg) and Zn (49 mg/kg) [50]. Levels of Zn were also much higher in Kuils River than those observed in a Ugandan study, in which Namuhani et al. [1] reported a mean concentration of 28 mg/kg around a steel mill in that country.

According to Igeo, the heavy metals that appeared to be of particular concern were As (moderately to extremely contaminated), Pb and Sr (uncontaminated to heavily contaminated) and Zn (moderately to extremely contaminated). Two of the three preschools extremely contaminated by Zn are within a radius of 500 m from the industrial belt, particularly close to the steel manufacturing industry. Although the one school extremely contaminated by Sr is not particularly close to the Kuils River industrial belt, it must be noted that it is close to a landfill site, a wastewater plant and a secondary industrial zone which includes galvanizers and steel producers. Compared with findings from studies conducted in Uganda [1], China [47] and elsewhere in South Africa [6,24], there are indications that preschool institutions in the Kuils River study site may be exposed to somewhat elevated concentrations of certain heavy metals in soil from anthropogenic and natural sources.

The use of factor analysis and the Pearson correlation coefficient to identify the sources of heavy metals showed that the heavy metals were well represented by the first two factors, accounting for more than 80% of the total variance. As, Mn and Sr were significantly correlated and highly loaded on Factor 1, indicating common sources [34]. As–Mn–Sr are known to result from smelting operations, atmospheric deposition from fossil fuel combustion, aluminum alloys, paint etc. [47]. However, according to Igeo, EF and CF results, the preschools were not contaminated with Mn. In this light, it is possible that the sources of heavy metals could be loaded with a combination of industrial operations and natural sources for Factor 1 [1,47]. Pb, Fe and Zn were significantly correlated and highly loaded on Factor 2, also indicating common sources [51]. Although these heavy metals are regarded as marker elements of industrial operations [1,47,48], Pb is also a marker for non-exhaust vehicle emissions (i.e., tire wear, braking system) [52], and Zn is a marker for petrochemical, fossil fuels and other industrial activities [47]. According to Igeo, EF and CF results, the preschools were uncontaminated with Fe. Therefore, Factor 2 could be a mixture of industrial operations, non-exhaust vehicle emissions and natural sources [1,47,48].

Non-carcinogenic effects were significant in our study, and the carcinogenic risk was also observed. Similar results were found in previous studies for soil [24] in urban environments. For example, in Johannesburg, South Africa an HI value >1 (which poses serious non-carcinogenic effects) was estimated among children living in the gold mining area. The carcinogenic risk was found to be 3.67 × 10^−4^, implying that of 2725 children, one child may be affected [24].

This was a baseline study following public complaints of adverse health outcomes as a result of the steel mill in Kuils River. Studies have shown decreasing metal levels with increasing distance from industrial operations, including steel mills [53]. In this study, it did not appear that the levels of Fe, Sr and Mn decreased as the distance from the steel mill increased. These results support our Igeo, EF and CF analysis, which indicated that the study area was not contaminated with Fe and Mn. In relation to Sr, it is possible that meteorological conditions influenced the deposition of heavy metals in the soil, as reported in the literature [54]. The median levels of As, Pb, Ti and Zn within 2 km of the industrial zone were, however, higher than the heavy metal levels further away. Further, according to Getis Ord Gi* hot spots, all heavy metals were mainly distributed between 0.3–2.2 km from the industrial zone (from an easterly to westerly direction). These findings are comparable to other studies in the literature that have shown higher pollutant levels in residential homes situated in close proximity to industrial operations, compared to homes further away [5,47,55]. The steel mill was established in the 1960s and operated until 2010. In 2018 it was reopened [56]. Although the steel mill was not operating at the time of data collection, it had since been reopened.

Alongside emerging evidence from research undertaken elsewhere in South Africa, the findings from the current study point to a need for greater care in planning and decision making vis-à-vis the location of human settlements in relation to industrial locations. A good starting point may be to conduct risk and health impact assessments for major new developments to inform decisions on community health protection and promotion. Buffer zones between sites of pollution and human settlements represent one example from a suite of interventions that may be implemented to help protect the health of communities; others include developing or implementing emission standards and regulations for industries, public transportation expansion, etc. With regards to buffer zones, identifying the optimal protective distance from the pollutant-generating activities is complex, given the variety of heavy pollutants emitted by industrial operations, transportation of pollutants in the environment, local climatic and weather conditions, and the severity of health outcomes [57]. In South Africa, the environment is protected by the National Environmental Management Act; however, there is insufficient epidemiological data to inform decisions on appropriate buffer zones from a health perspective.

This baseline study provides evidence of heavy metal exposure in a vulnerable community situated in close proximity to pollutant generating operations. It indicates a missed opportunity to implement healthy planning practices that might have averted exposure to heavy metals and other pollutants in the longer term, and the associated burden of ill health for resident communities as well as the future cost of care that will be borne by the health sector and the economy overall.

One of the study limitations was that a convenience sample of the preschools situated around the industrial operation was drawn, and although all the preschools situated within 5 km of the industrial operation were targeted, only 34 preschools could be included in the study (e.g., some of the preschools were closed down due to COVID-19 restrictions). Previous research found a high correlation between heavy metals from similar sources [47]. Our results also suggest similar natural and anthropogenic sources for heavy metals, however, some of the heavy metals showed non-significant correlations, demonstrating a variability among the sources of origin. Therefore, future research should focus on evaluating various sources of heavy metals within the study area. The hand-to-mouth behavior in children may play a role in elevated blood metal distributions [58]. Future research is needed to elucidate children’s exposure to heavy metals, including surveys of children’s blood, hair and urine, environmental samples (i.e., soil, air, water), and health outcomes. The current study provides a baseline against which the impacts of further operation of polluting industries on soil metal concentrations might be measured.

## 5. Conclusions

This study provides evidence of exposure to heavy metals in a vulnerable group—preschool children. Although metal concentrations in soil samples were below the South African reference levels, at certain preschools, As and Zn concentrations were found to exceed Canadian soil reference levels. Factor analysis indicated contributions from industrial operations, non-exhaust vehicular emissions and natural sources to heavy metal exposure in the study population. Our findings indicated that the ingestion pathway was the greatest contributor to the non-carcinogenic risk. The ingestion pathway also contributed the most to cancer risk. The dermal and inhalation pathways contributed the least to risk. The study findings may be used to lobby for application of the precautionary principle in environmental health with regard to the location of polluting industries vis-à-vis human settlements, and as a baseline against which future studies of environmental contamination in the Kuils River area may be measured.

## Figures and Tables

**Table 1:** Reference doses (mg/kg/day) and cancer risk factors for heavy metals.

	RfDing	RfDInh	RfDderm	CSFing	CSFinh	CSFderm	Refs.
As	3.0 × 10^−4^	3.0 × 10^−4^	3.0 × 10^−4^	1.5 × 10^0^	1.5 × 10^0^	1.5 × 10^0^	[32,41]
Fe	8.4 × 10^0^	2.2 × 10^−4^	7.0 × 10^−2^	NF	NF	NF	
Mn	140 × 10^−3^	1.4 × 10^−3^	1.8 × 10^−3^	NF	NF	NF	[35,42]
Pb	3.6 × 10^−3^	1.4 × 10^−3^	1.4 × 10^−3^	8.5 × 10^−3^	4.2 × 10^−2^	4.2 × 10^−2^	[32,43]
Sr	6 × 10^−1^	NF	NNF	NF	NF	NF	
Ti	NF	NF	NF	NF	NF	NF	
Zn	3.0 × 10^−1^	3.0 × 10^−1^	7.5 × 10^−2^				[32,44]

NF: Not found.

**Table 2:** Summary of heavy metal levels in soil (*n* = 34).

mg/kg	Arsenic (As)	Iron (Fe)	Manganese (Mn)	Lead (Pb)	Strontium (Sr)	Titanium (Ti)	Zinc (Zn)
Min	9	1143	100	16	35	441	149
Max	28	16116	256	97	834	4378	834
Mean (SD)	16 (±17)	4469 (±3093)	137 (±40)	30 (±18)	176 (±16)	1547 (±765)	232 (±123)
Median	15	3838	126	24	212	1324	204
* Background values	1.5	9800	1275	10.5	30	1500	142.5
** % > South African reference levels	0%	NRL	0%	0%	NRL	NRL	0%
*** % > Canadian reference levels	31%	NRL	0%	0%	NRL	NRL	9%
% > sample mean	36%	50%	31%	22%	33%	38%	21%

* The background values for the study area were based on Turekian and Wedepohl [26]. ** The South African reference levels for As, Mn, Pb and Zn are 48 mg/kg, 1500 mg/kg, 230 mg/kg and 19 000 mg/kg, respectively [24]. *** The Canadian reference levels for As, Mn, Pb and Zn are 18 mg/kg, 740 mg/kg, 120 mg/kg and 290 mg/kg, respectively [23]. NRL: No reference level.

**Table 3:** Z-score values for heavy metals at preschools in relation to the industrial zone.

* Preschool ID	lgeo	** Gi-z-Score	** Gi-*p*-Value	Distance from Industrial Zone	Direction from Industrial Zone
E	Ti	−2.293	0.021	2.19	West
F	Ti	2.062	0.039	0.94	West
G	Ti	2.001	0.045	0.40	West
A	Ti	2.001	0.045	0.32	West
D	Fe	3.395	0.0007	0.46	East
C	Fe	2.838	0.005	0.51	East
E	Fe	−2.092	0.036	2.19	West
C	Mn	2.359	0.018	0.51	East
B	Mn	2.003	0.045	1.51	West
D	Pb	3.475	0.0005	0.46	East
C	Pb	2.793	0.005	0.51	East
H	As	2.371	0.018	2.32	Northeast
D	As	2.139	0.032	0.46	East
A	Zn	2.446	0.014	0.32	West
G	Zn	2.446	0.014	0.40	West
F	Zn	2.063	0.039	0.94	West

* For anonymity, preschool ID is the unique identifier assigned to each preschool. ** The z-scores and *p* values indicate if features show statistically significant clustering or dispersion. ** Significant negative z-scores indicate that there is a clustering of low values (characterized as a cold spot). Significant positive z-scores indicate that there is a clustering of high values (characterized as a hot spot).

**Table 4:** Factor analysis results of heavy metal levels in soil.

Metal	Factor 1	Factor 2
As	**0.908**	0.226
Fe	0.348	**0.844**
Mn	**0.777**	0.289
Pb	0.107	**0.870**
Sr	**0.969**	0.083
Zn	0.092	**0.443**
Ti	0.244	0.249
Eigenvalues	4.2	1.5
Proportion of variance (%)	61.1	22.2
Cumulative (%)	61.1	83.3

Bolded values show heavy metals in each factor that have communalities >40%. Heavy metals with values below <40% were excluded for each factor.

**Table 5:** Matrix of correlation coefficients among heavy metals.

Metal	As	Fe	Mn	Pb	Sr	Ti	Zn
As	1	0.75 ***	0.75 **	0.45	0.51 *	0.62 **	0.45
Fe		1	0.41	0.68 ***	−0.24	0.83 ***	0.53 ***
Mn			1	0.33	0.53 *	0.33	0.47
Pb				1	0.19	0.45 *	0.68 ***
Sr					1	−0.27	0.19
Ti						1	0.64 ***
Zn							1

*** Correlation is significant at the 0.001 level. ** Correlation is significant at the 0.01 level. * Correlation is significant at the 0.05 level.

**Table 6:** Estimated average daily intake (ADI), hazard quotient (HQ), hazard index and cancer risk for lifetime exposure (LCR).

	ADIing	ADIinh	ADIderm	HQing	HQInh	HQderm	HI	LCR
As	1.8 × 10^1^	7.0 × 10^−10^	2.3 × 10^−6^	6.1 × 10^4^	2.3 × 10^−6^	7.8 × 10^−3^	6.1 × 10^4^	2.7 × 10^1^
Pb	3.4 × 10^1^	1.3 × 10^−9^	4.4 × 10^−6^	9.5 × 10^3^	5.3 × 10^−7^	1.2 × 10^−3^	1.0 × 10^4^	2.9 × 10^−1^
Fe	6.0 × 10^4^	2.3 × 10^−6^	7.6 × 10^−3^	7.1× 10^3^	1.0 × 10^−2^	1.1 × 10^−1^	7.0 × 10^3^	NC
Mn	2.0 × 10^3^	7.0 × 10^−8^	2.3 × 10^−4^	1.3 × 10^6^	5.0× 10^−5^	1.3× 10^−1^	1.3 × 10^6^	NC
Sr	2.0 × 10^3^	9.0 × 10^−8^	3.0 × 10^−4^	NC	NC	NC	NC	NC
Ti	2.1 × 10^4^	7.9 × 10^−7^	2.6 × 10^−3^	NC	NC	NC	NC	NC
Zn	3.0 × 10^3^	1.2 × 10^−7^	4.0 × 10^−4^	1.0 × 10^4^	4.0 × 10^−7^	5.3 × 10^−3^	1 × 10^4^	NC

NC: not calculated.

## Data Availability

The data that support the findings of this study are available on request from the corresponding author. The data are not publicly available due to privacy or ethical restrictions.
